# Notes upon Somnal, the New Hypnotic

**Published:** 1891-01

**Authors:** Frank Woodbury

**Affiliations:** Fellow of the College of Physicians of Philadelphia; Hon. Professor of Clinical Medicine in the Medico-Chirurgical College, etc.


### NOTES UPON SOMNAL, THE NEW HYPNOTIC.

By FRANK WOODBURY, A. M., M. D.,

Fellow of the College of Physicians of Philadelphia; Hon. Professor of Clinical Medicine in the Medico-Chirurgical College, etc.

Last Fall, Radlauer[1] of Berlin brought to the notice of the medical profession a new compound to which he gave the name of Somnal, in acknowledgment of the remarkable hypnotic properties which it appeared to possess. It was formed by the union of chloral, alcohol and urethane, according to the original notice,[2] but is not a simple mixture of these bodies. It differs from chloral-urethane by the addition of $C^2H^4$, its formula being $C\ H^{12}Cl^3O^3N$. The method of manufacture is by direct combination of chloral alcoholate and urethane in a vacuum apparatus, according to its discoverer, who states[3] that its composition might be graphically represented thus:

$$

\begin{array}{l}

\qquad\qquad OC^2H^5 \\

CCl^3{-}C{-}H \\

\qquad\qquad NHCOOC^2H^5.

\end{array}

$$

Specimens of this new hypnotic having, through the courtesy of Messrs. Eisner & Mendelson Co., been placed in my hands for examination and trial, I will here, very briefly, communicate some of the results thus far obtained, reserving my final judgment upon the drug until experience has been more extended.

*Physical Characters.*—Somnal is a colorless liquid, resembling chloroform in its appearance and behavior when added to cold water, in which it forms globules and refuses to mix or dissolve. When shaken with water, the mixture is milky but quickly separates. It is soluble in hot water and alcoholic solutions, and dissolves resinous substances and fats. The odor is faint, not very

1. Zeitschrift des Apothekers-Vereins, Nov., 1889.
2. Journal de Médecine, Oct. 20, 1889.
3. *Pharmaceutical Journal and Transactions*, Nov., 1889.

penetrating or disagreeable, and resembles that of the spirits of nitrous ether, or recrystalized chloral. The taste is very pungent; and for administration it needs free dilution. It may be given with whisky or solution of tincture of zingiber, or syrup of licorice. Somnal is inflammable, burning with an alcoholic flame; it does not evaporate quickly, and leaves a greasy stain upon blotting paper. Specific gravity greater than water; reddens litmus paper slightly.

*Physiological Effects.*—In its action it resembles chloral in quickness of effect and naturalness of the sleep produced. No marked depressing influence was exerted upon the pulse or respiration rate, though it was noticed that the breathing became slower and the pulse slower and fuller as in natural repose. No disagreeable after-effects. The head was clear and the stomach was unaffected; the patients generally had an appetite for breakfast. No constipating effect. The kidneys acted rather more freely than usual. My colleague, Dr. Ernest Laplace, to whom I gave some of the drug for trial at the Philadelphia Hospital, writes as follows:

"I have given somnal a fair trial upon six patients at the Philadelphia Hospital. In no case were the patients told what was given them, so outside of the bare possibility of the patients' falling asleep through natural causes, somnolence was brought on by the drug. It was administered in a solution of tinct. zingiberis, in half-teaspoonful doses, and was found palatable.

"Administered at 4 P. M., at a moment when patients were not generally asleep, in four cases sleep came on within half an hour, which lasted from five to eight hours; the two other cases showed no effect from the drug. It is their habit to get at least gr. $\frac{1}{4}$ of morphine sulph. to put them asleep every night, as they are sufferers from intractable malignant growth.

"In no case was there any noticeable after-effect.

"I have not formed any opinion upon the length of time that the drug could be used daily upon the same patient.

"To this I might add that no depression of the normal temperature was noticed in any case in my hands, and thus far I have not used it in pyrexia."

*Therapeutic Application.*—The effects of somnal in producing natural sleep suggested its use in insomnia. The first case in which I used it was in a patient suffering with acute alcoholism, who had been under treatment for a fortnight in an institution where he had a free supply of liquor, and he came out rather worse than he went in. He was thirty-nine years of age, very tremulous, and could not sleep, or if he dozed off would immediately waken up. I gave him, at about 3 P. M., thirty minims of somnal (or rather a drachm of a mixture of equal parts of somnal and whisky), well diluted, and went into an adjoining room to speak to an attendant. Upon my return I was surprised to find him fast asleep, although I had not been away from him more than fifteen minutes. He slept for four hours, and then was able to take something to eat. At ten o'clock he had another dose, and he slept until seven the next morning, having wakened up once only during the night, and insisted upon having another dose, and immediately after taking it he fell asleep again. The next night he was given a double dose at 10 P. M., and he slept all night without wakening. No bad effects were observed. The somnal was given for four nights, when he was so nearly well that it was suspended, as he had had good natural sleep at night and seemed quite restored. Alcohol was positively prohibited, the only substitute allowed being Elixir of Coca and Camellia (P. D. & Co.), in tablespoonful doses, in which it is true there was a small amount of alcohol, which was quite infinitesimal when compared with what he had been using. Somnal, therefore, acts well as a hypnotic in acute alcoholism as a tranquillizer and hypnotic.

In a case of neuralgia of the bowels (visceral neurosis of Allbutt), where the patient had a sleepless night, a dose of twenty minims relieved nausea and pain, and the patient fell asleep.

In syphilitic headache and insomnia, somnal, in moderate doses, failed to produce sleep, which was afterwards secured by potassium bromide and iodide, and antipyrin.

In cases of insomnia, fretfulness, and restlessness in young children, somnal with mint water and syrup offers better results than opiates, and is much safer. The same remark probably applies to the use of somnal in acute pneumonia, but I have not been able to confirm this yet by actual trial.

Without further going into detail it may be stated in conclusion that somnal acts as a hypnotic, but instead of depressing the system as chloral does, it slightly stimulates the gastric mucous membrane, relieves nausea and pain, improves the appetite, increases secretion (probably), does not cause constipation. The circulation, respiration, and temperature are not notably depressed after its administration. No disagreeable after-effects have been observed. As it is rapidly eliminated from the body, it may be administered each night for a number of days without any obvious ill-effects. It acts very much like chloral, but is more pleasant to take, and not so depressing in its effects upon the nervous system and the circulation.—*The Dietetic Gazette.*

